# Structural transition of replicable RNAs during in vitro evolution with Qβ replicase

**DOI:** 10.1261/rna.073106.119

**Published:** 2020-01

**Authors:** Ryo Mizuuchi, Kimihito Usui, Norikazu Ichihashi

**Affiliations:** 1 Komaba Institute for Science, The University of Tokyo, Meguro, Tokyo 153-8902, Japan; 2 JST, PRESTO, Kawaguchi, Saitama 332-0012, Japan; 3 Japan Science and Technology Agency, Suita, Osaka, 565-0871, Japan; 4 Department of Life Science, Graduate School of Arts and Science, The University of Tokyo, Meguro-ku, Tokyo, 153-8902, Japan; 5 Universal Biology Institute, The University of Tokyo, Meguro-ku, Tokyo, 153-8902, Japan

**Keywords:** RNA secondary structure, single-stranded RNA, replication, evolution, Qβ replicase

## Abstract

Single-stranded RNAs (ssRNAs) are utilized as genomes in some viruses and also in experimental models of ancient life-forms, owing to their simplicity. One of the largest problems for ssRNA replication is the formation of double-stranded RNA (dsRNA), a dead-end product for ssRNA replication. A possible strategy to avoid dsRNA formation is to create strong intramolecular secondary structures of ssRNA. To design ssRNAs that efficiently replicate by Qβ replicase with minimum dsRNA formation, we previously proposed the “fewer unpaired GC rule.” According to this rule, ssRNAs that have fewer unpaired G and C bases in the secondary structure should efficiently replicate with less dsRNA formation. However, the validity of this rule still needs to be examined, especially for longer ssRNAs. Here, we analyze nine long ssRNAs that successively appeared during an in vitro evolution of replicable ssRNA by Qβ replicase and examine whether this rule can explain the structural transitions of the RNAs. We found that these ssRNAs improved their template abilities step-by-step with decreasing dsRNA formation as mutations accumulated. We then examine the secondary structures of all the RNAs by a chemical modification method. The analysis of the structures revealed that the probabilities of unpaired G and C bases tended to decrease gradually in the course of evolution. The decreases were caused by the local structural changes around the mutation sites in most of the cases. These results support the validity of the “fewer unpaired GC rule” to efficiently design replicable ssRNAs by Qβ replicase, useful for more complex ssRNA replication systems.

## INTRODUCTION

The replication of genetic information is a fundamental function of living systems. Single-stranded RNA (ssRNA), one of the possible genetic information carriers, is used as a genomic molecule in some viruses and also experimental models of primitive life-forms in the hypothetical RNA or RNA–protein world (Higgs and Lehman 2015; Joyce and Szostak 2018). The synthesis and evolution of such experimental models provide important insights into the early evolution of life (Szostak et al. 2001; Ichihashi 2019).

To date, important information regarding ssRNA replication has been obtained from studies using Qβ replicase, the RNA replicase of bacteriophage Qβ (Takeshita et al. 2014; Gytz et al. 2015; Chetverin 2018). One of the most serious problems for ssRNA replication is double-stranded RNA (dsRNA) formation, which occurs through the association between a template and a newly synthesized strand during replication (Axelrod et al. 1991; Usui et al. 2015; Chetverin 2018). DsRNA is a dead-end product for usual ssRNA replication systems because large energy is required to dissociate a dsRNA. A possible method to overcome this problem is to use ssRNA that is likely to form intramolecular base-pairings (i.e., secondary structures), which hinder intermolecular base-pairings (i.e., double-strand formation). The design of such an ssRNA sequence is possible by making secondary structures throughout the sequence and decreasing unpaired G and C nucleotides, which facilitate the association of the ssRNA with the complementary strand (Usui et al. 2015). The importance of secondary structure and this “fewer unpaired GC rule” has been supported by the fact that we have successfully created replicable RNAs of <800 nt by following this rule (Usui et al. 2015; Mizuuchi and Ichihashi 2018). However, there is limited evidence for the applicability to longer ssRNAs, which can encode any genes for broader applications (Ueda et al. 2019).

To examine the validity of the rule for a longer RNA from a different aspect, here we analyzed mutations introduced during an in vitro evolution of replicable ssRNAs. Through in vitro evolution, mutations should be successively introduced in the order of effectiveness to improve replication if population size is sufficiently high. Therefore, if the introduced mutations during the evolution follow the rule, it should strongly support the primary importance to obey the “fewer unpaired GC rule” for efficient replication. We recently performed an in vitro evolution of an artificial genomic ssRNA that encodes Qβ replicase (2125 nt) in a reconstituted translation system of *Escherichia coli* encapsulated in microscale water-in-oil droplets (Ichihashi et al. 2013). During the evolution, the RNA evolved to be more efficiently replicated by the replicase with less dsRNA formation. In this experiment, the population size of ssRNA was sufficiently high (∼10^8^), which presumably covers all possible point mutations. We believe that a series of RNAs that appeared in the course of the evolutionary experiment would be ideal samples to evaluate the importance of the rule for ssRNA replication.

In this study, we focused on one of the main evolutionary routes, consisting of nine distinct genotypes that successively accumulated 11 mutations. First, we found that the replication ability increased step-by-step by decreasing the probability of dsRNA formation. We then examined secondary structures of all the nine ssRNAs using a structure-dependent chemical modification data. From the structural data, we found that the numbers of unpaired G and C bases were gradually decreased as mutations accumulated. These results support the importance of the fewer unpaired GC rule for efficient ssRNA replication by Qβ replicase.

## RESULTS

### RNAs used in this study

**FIGURE 1.**
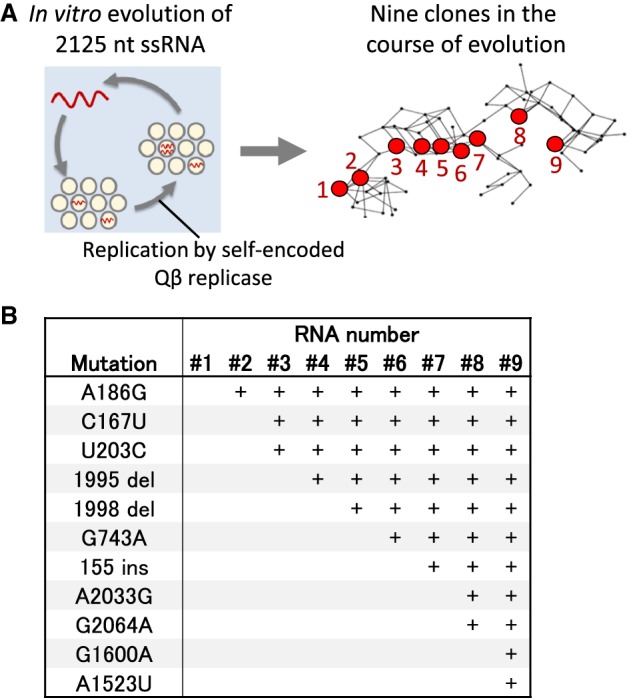
Nine RNAs analyzed in this study. (*A*) Scheme of the evolutionary experiment and positions of the nine analyzed RNAs in a genetic map. In vitro evolution of an ssRNA was performed in a previous study (Ichihashi et al. 2013) in which an ssRNA replicated by self-encoded Qβ replicase in microscale droplets (*left*). During the evolution, various genotypes of the RNA appeared, and the relationships of dominant genotypes (existed in more than 1% of the population) in a given stage of the evolution are represented on a two-dimensional sequence map (Ichihashi et al. 2015). Each genotype is plotted as a point while maintaining mutual genetic distance, in which red ones indicate the RNA clones analyzed in this study (*right*). Genotypes with one mutation apart are connected by a line. Note that we did not observe direct intermediate genotypes that connect RNA #7 with #8 and #8 with #9 by one mutation. A detailed map is shown in Supplemental Figure S1. (*B*) The list of mutations introduced in the nine RNAs. The mutations “1995 del” and “1998 del” represent the deletion of three bases at each position. The mutation “155 ins” represents the insertion of six bases at the position. The numbers reflect the base number in the original RNA #1.

In our previous study, we performed an in vitro evolutionary experiment using ssRNA (2125 nt) that encoded the catalytic subunit of Qβ replicase in a reconstituted translation system of *Escherichia coli* (Shimizu et al. 2001; Ichihashi et al. 2013). In this experiment, the ssRNA was replicated by the self-encoded replicase and then amplified through reverse-transcription, PCR, and in vitro transcription. Mutations were spontaneously introduced during both the replication and the amplification processes. When we continued this translation-coupled RNA replication for 32 rounds in microscale water-in-oil droplets (Fig. 1A left), mutant RNAs with higher replication ability appeared and dominated the population repeatedly. We have sequenced the RNA populations and found that 91 different genotypes that consist of the combination of 22 mutations appeared during the evolution (Ichihashi et al. 2015). In this study, we focused on nine RNA genotypes (#1–#9) that comprise one of the main evolutionary routes (Fig. 1A right; Supplemental Fig. S1). In this evolutionary route, the nine RNA genotypes successively accumulated 11 types of mutations, including point mutations, an insertion, and two deletions, by one or two mutations at each transition (Fig. 1B). The 11 types of mutations covered most (11 out of 16) of the fixed mutations introduced in the genotypes shown in Figure 1A.

**FIGURE 2.**
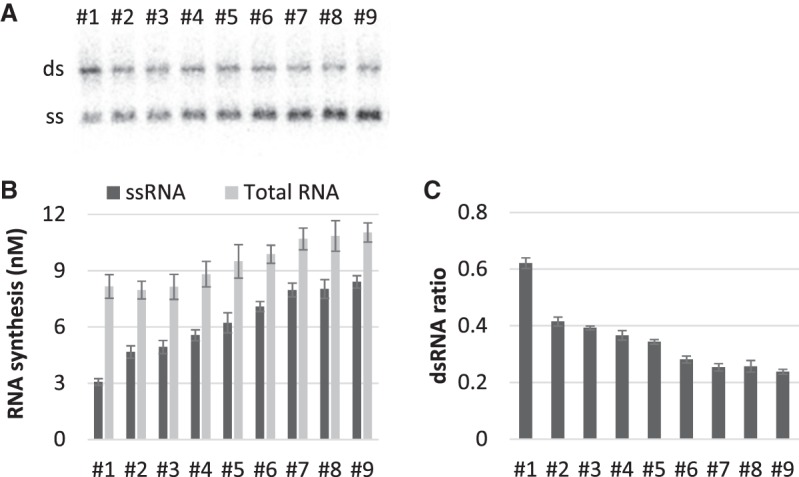
Template abilities of the RNAs. (*A*) Each RNA (100 nM) was incubated with purified replicase (20 nM) at 37°C for 20 min, and synthesized RNA concentrations were measured by [^32^P]-UTP incorporation assay followed by agarose gel electrophoresis and autoradiography as previously described (Usui et al. 2013). An example of a gel image is shown; “ds” and “ss” represent dsRNA and ssRNA, respectively. (*B*) Synthesized ssRNA and total RNA (sum of ss- and dsRNA) concentrations. (*C*) dsRNA ratio in synthesized total RNA. Note that in principle, some dsRNA could have formed after the replication reaction, such as through electrophoresis. Error bars show the standard errors of four independent experiments.

The RNAs that appeared during the evolutionary experiment have two types of replication ability: the ability of the encoded replicase (replicase ability) and the ability to be replicated as a template (template ability). In the previous study, we measured these activities for some RNAs that finally dominated the population and found that the template ability, rather than the replicase ability, selectively improved during the evolution (Ichihashi et al. 2013). To evaluate the template abilities of the nine ssRNAs, not measured in the previous study, we performed replication of each of the nine ssRNAs with a purified Qβ replicase and measured the replicated ssRNA concentration, together with the ratio of dsRNA formation in the RNA products, by [^32^P]-UTP incorporation assay followed by agarose gel electrophoresis and autoradiography (Fig. 2A). In all the transitions except for one (RNAs #7 to #8), the ssRNA synthesis by replication of the clone RNAs increased step-by-step (Fig. 2B) as mutations accumulated, whereas the dsRNA ratios in synthesized total RNA products (ssRNA and dsRNA) were gradually decreased (Fig. 2B,C). These results indicate that RNA sequences that can efficiently replicate as ssRNAs were selected in this evolutionary route.

### Global structure analysis

To investigate structural changes in the nine RNAs, we performed selective 2′-hydroxyl acylation analyzed by primer extension (SHAPE) analysis (Merino et al. 2005). First, we chemically modified unpaired bases of the ssRNAs and then conducted reverse-transcription to produce cDNAs. Because reverse-transcription stopped at modified bases, we obtained base-pairing probability at each base from the size distribution of the cDNA analyzed by capillary electrophoresis. If the SHAPE value of a base is close to zero, the base is likely to form a base pair, whereas if the SHAPE value is close to one, the base is likely not. We performed SHAPE analysis three times and obtained SHAPE values for more than 90% of the nine RNA sequences (Supplemental Fig. S2). The average SHAPE value and standard deviation for each RNA are shown in the Supplemental information.

**FIGURE 3.**
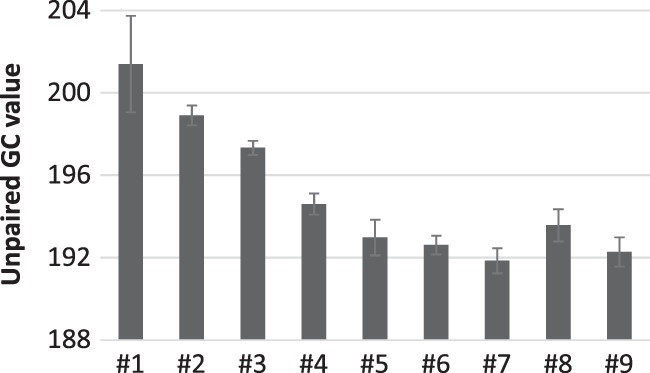
Unpaired GC values of the entire sequences. The unpaired GC values of the entire sequences of RNA #1 to #9 were calculated using the average SHAPE values as described in the Materials and Methods section. The values represent the sum of the probability that each G or C nucleotide is unpaired throughout the RNA sequences. Error bars represent standard errors of unpaired GC values calculated from three independent sets of SHAPE values.

To investigate the validity of the “fewer unpaired GC rule,” we used these average SHAPE values to obtain partition functions of the nine clone RNAs by using RNAstructure software (Mathews 2014), which provides all possible canonical base-pair probabilities throughout each sequence. We then calculated “unpaired GC value,” defined as the sum of the probability that each G or C nucleotide is not base-paired over the entire sequence. The probability at each nucleotide is at most one, and therefore the maximum possible unpaired GC value equals the number of investigated nucleotides. We found that the unpaired GC value for the entire sequence of each clone decreased at all transitions from RNA #1 to #9 except for the step from RNA #7 to #8 (Fig. 3). Given that the template ability did not increase at the transition from RNA #7 to #8 (Fig. 2), the decrease in unpaired GC value is consistent with the increase in the template abilities (see also Supplemental Fig. S3 for direct comparison between the unpaired GC values and template abilities). These results indicate that the improved template abilities of the nine RNAs can be understood based on the fewer unpaired GC rule.

**FIGURE 4.**
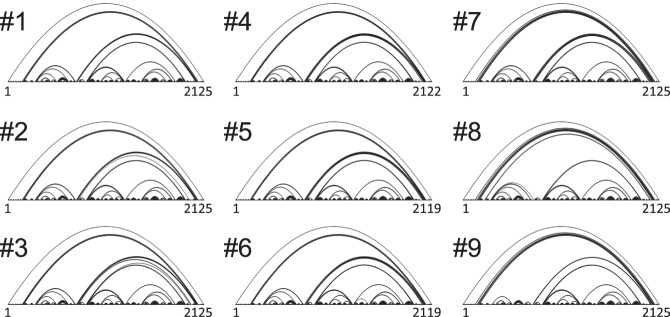
Base-pairing pattern of MEA structures. MEA structures were predicted by using a software (RNAstructure) with a constraint of SHAPE values shown in Supplemental Figure S2 and represented as arch diagrams. The horizontal line at the *bottom* of each diagram represents each RNA sequence from *left* to *right*. The curved lines represent base pairs of the connected bases.

Next, to investigate how the structures changed to reduce unpaired GC values, we predicted the maximum expected accuracy (MEA) structures from the partition functions by using the same RNAstructure software (Mathews et al. 2009; Mathews 2014). The base-pairing patterns of the MEA structure were shown as arch diagrams (Fig. 4). The overall patterns were similar for all structures, indicating that no significant global structural change occurred during the evolution. Detailed MEA structures are shown in bracket format in Supplemental Text. In the following part of this study, we analyzed these MEA structures.

### Local structural changes around the mutation sites

**FIGURE 5.**
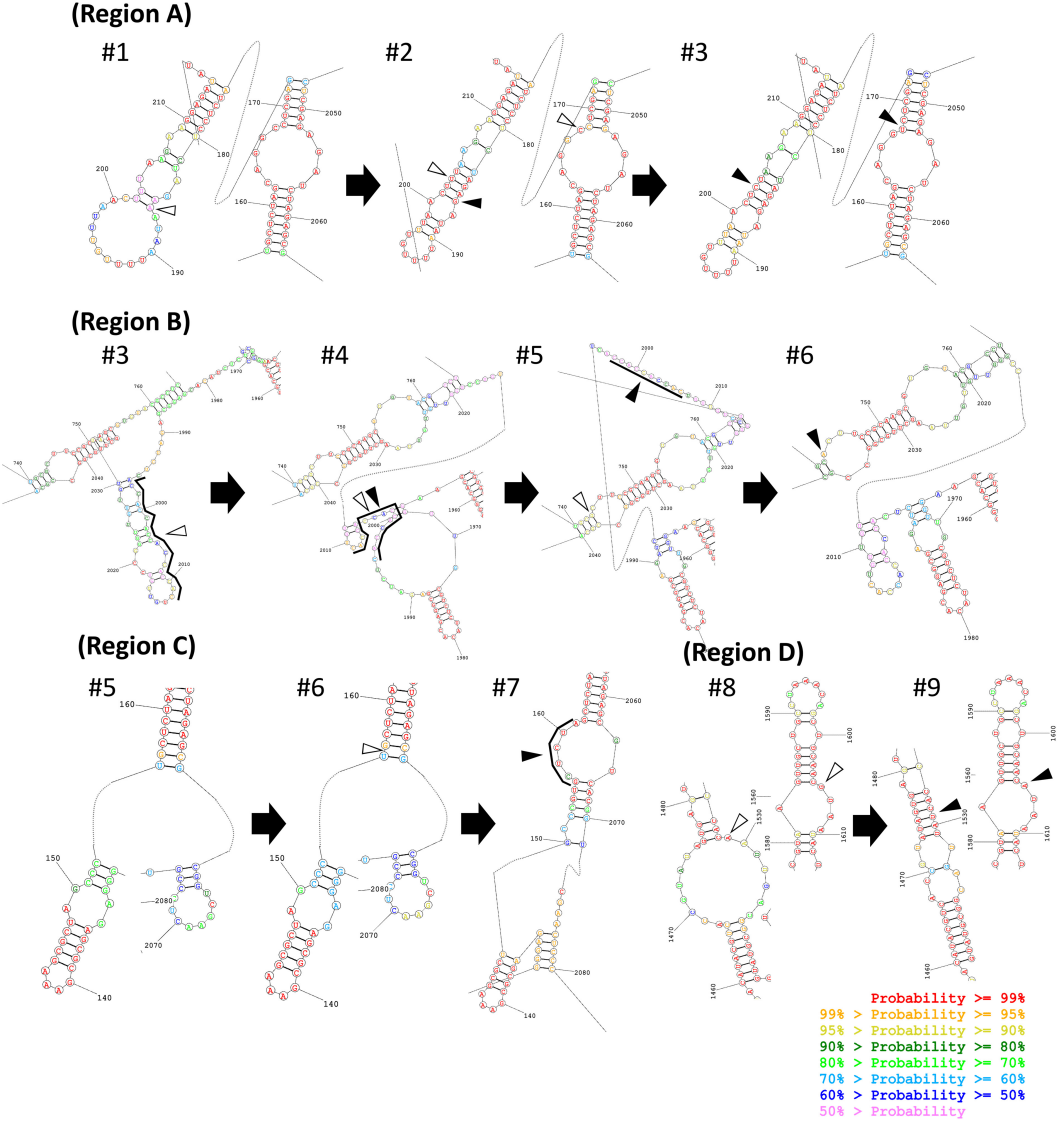
Local MEA structures around the mutation sites. Filled and open triangles indicate the sites where mutations were introduced or will be introduced in the next RNA, respectively. Note that mutation sites cannot be uniquely determined at transitions from RNA #3 to #4 and from RNA #4 to #5, at which one of the tandem repeats of “CAU” (indicated with bold lines) was deleted. At the transition from RNA #5 to #6, “GCUCUA” was inserted at the region indicated with a bold line. Dotted lines represent zero to three consecutive unpaired nucleotides. Nucleotide colors reflect prediction accuracy for each base as shown in the *bottom right*.

We next investigated how each introduced mutation contributes to the reduction in the unpaired GC value by examining the local structures around mutation sites. Notably, mutations introduced in the nine RNAs are located in four local structural regions, named regions A (base numbers 156–215, 2047–2063), B (740–765, 1965–2046), C (130–155, 2064–2080), and D (1454–1606) (Fig. 5), which contain mutations introduced in RNA #2–3, #4–6, #7, and #9, respectively. The mutational sites before and after mutation are indicated by open and filled arrowheads, respectively. Regions A, B, and C are adjacent, and the entire structure including these regions is shown in Supplemental Figure S4.

**FIGURE 6.**
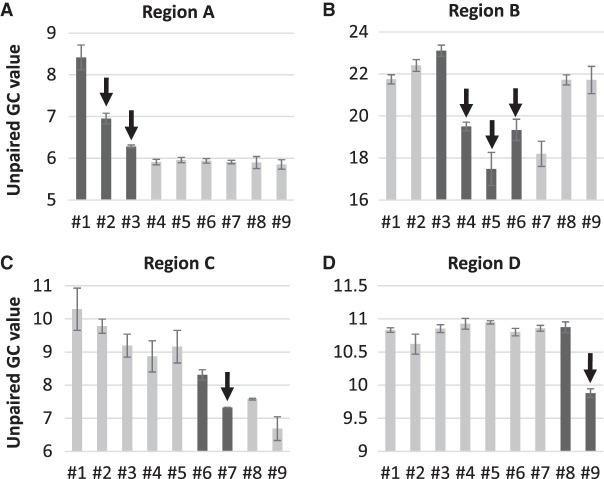
Unpaired GC values of the local MEA structures around mutation sites. Unpaired GC values were calculated for regions A (base numbers 156–215 and 2047–2063), B (740–765 and 1965–2046), C (130–155 and 2064–2080), and D (1454–1606). Error bars represent standard errors of unpaired GC values calculated from three independent sets of SHAPE values. The arrows indicate the points where mutations were introduced.

We then examined each transition of these local structural regions in detail. At the transition from RNA #1 to #2, the introduced mutation (A186G) induced base-pairings around the mutation site, which decreased the probability of unpaired G and C bases in region A, and hence reduced unpaired GC value (Fig. 6A). At the transition from RNA #2 to #3, the mutation (C167U) was introduced in an unpaired site and thus directly reduced the probability of unpaired C. In these transitions, the introduced mutations affected the base-pairing pattern close to the mutation sites, whereas at the transitions from RNA #3 to #5, the introduced mutations changed the local base-pairing patterns drastically to decrease the unpaired GC value of this region as a whole (Fig. 6B). At the transition from RNA #5 to #6, the local unpaired GC value of the region B, which contains the mutation site, increased (Fig. 6B), whereas in the adjacent region C, the base-pairing pattern was retained, but the overall accuracies of each base-pairing changed to decrease the unpaired GC value (Fig. 6C). This result implies that the reduction of the unpaired GC value occurred in a distant region from the mutation site at this transition. At the transition from RNA #6 to #7, the insertion of six bases in region C drastically changed the local structures and reduced the unpaired GC value in the region (Fig. 6C). At the transition from RNA #8 to #9, the mutation (G1600A) introduced in an unpaired G directly decreased the unpaired GC value in the region (Fig. 6D). In summary, the introduced mutations decreased the local unpaired GC values around the mutation sites in most of the transitions.

## DISCUSSION

In this study, we analyzed the step-by-step evolution of RNA structures for ssRNA replication by Qβ replicase. Focusing on nine previously obtained RNAs, we first showed that the RNAs gradually improved their template abilities with decreasing dsRNA formation as mutations accumulated (Fig. 2). Since the template ability depends on RNA structure, we analyzed the change of RNA structures during the evolution based on a secondary structure prediction algorithm with SHAPE data and found that the overall unpaired GC value decreased for all the evolutionary transitions at which replication abilities were increased (Fig. 3). These results suggest that the probability of unpaired G and C bases is an important factor to determine ssRNA replication by Qβ replicase, supporting the validity of “fewer unpaired GC rule” for a long RNA around 2125 nt.

One may think the unpaired GC value also correlates with other biophysical features of the RNA, such as overall folding stability. However, this should not necessarily be the case based on the suggested mechanism of dsRNA formation during replication (Usui et al. 2015) that unpaired G and C bases enhance interaction between loop structures of a template RNA and a complementary strand that is being synthesized. For example, even though a mutation that changes G or C in a loop structure to A or U without forming a base pair could reduce dsRNA formation, it hardly affects overall biophysical features such as folding stability. Actually, we calculated free energies and the number of base pairs for each MEA structure as an indication of biophysical features and found only a partial correlation with the unpaired GC values (Supplemental Fig. S5, correlation coefficients are −0.76 and 0.58, respectively), compared to the correlation between the ssRNA synthesis or dsRNA ratio and the unpaired GC values (Supplemental Fig. S3, correlation coefficients are −0.92 and 0.93, respectively).

Understanding the rule to efficiently design replicable long ssRNAs is particularly important to design synthetic ssRNAs that encode arbitrary genes. SsRNAs have been utilized as genomes of experimental models in the era of the RNA–protein world (Ichihashi et al. 2013), which provides important knowledge regarding possible evolutionary processes of a simple ssRNA genome (Ichihashi et al. 2015; Bansho et al. 2016; Mizuuchi and Ichihashi 2018). The design rule of ssRNA allows the development of such experimental models by introducing new genes, and thereby more complex biological phenomena can be demonstrated. Furthermore, this rule is potentially applicable to other replication systems using ssRNA such as ribozyme-based self-replication systems (Lincoln and Joyce 2009; Vaidya et al. 2012; Attwater et al. 2018), because the underlying mechanism of the “fewer unpaired GC rule” depends on the association between complementary RNA strands, and therefore it should not be limited to the replication by Qβ replicase. The “fewer unpaired GC” rule supported in this study paves the way for developing ssRNA-based replication systems.

This study also provides insights into the requirements for replicable RNAs by Qβ replicase. The effect of RNA structure on the replication by Qβ replicase has been extensively studied for decades (Mills et al. 1978; Axelrod et al. 1991; Biebricher and Luce 1993; Chetverin et al. 1997; Ugarov et al. 2003), but structures of long templates have rarely been investigated except for the native template, the genome of Qβ phage. In 2008, Ugarov et al. reported the importance of long-range interactions between termini for the overall rate of replication (Ugarov and Chetverin 2008). Here, we investigated the structures of nine RNAs that appeared during the evolutionary experiment and found approximately 2000-base long-range interactions between the termini for all the RNAs, and the addition of new base pairs at the transition from RNA #6 to #7 further stabilized it (Fig. 4), providing evidence to support the importance of long-range interactions.

It should be noted that the results of this study support the importance of the lower unpaired GC value for RNA replication by Qβ replicase, but it is one of many requirements to design an ssRNA that is efficiently replicable by Qβ replicase. Several other requirements are also known, such as 3′-terminal oligo C sequence (Kuppers and Sumper 1975), terminal stem structures (Biebricher and Luce 1993), uridine rich sequence (Brown and Gold 1995), and different structural requirements for illegitimate templates (Ugarov et al. 2003). Also, the S1 ribosomal subunit also enhances ssRNA replication by inhibiting the association of a template and its newly synthesized complementary strand (Vasilyev et al. 2013). The original RNA (RNA #1) we used here was constructed by inserting the β-subunit gene of Qβ replicase into a legitimate template, MDV-1 (Mills et al. 1973), and therefore it contains all the known requirements described above. In addition, in the in vitro evolutionary experiment, we used a reconstituted translation system of *E. coli* that contains approximately 3 µM of the S1 ribosomal subunit (Ichihashi et al. 2013).

To the best of our knowledge, this is the first study that analyzed the transition of RNA structures in the course of experimental evolution. The results presented here, therefore, provide important insights into a pattern of RNA structural changes during evolution. First, in most of the transitions, the local structures around the mutation sites primarily contributed to the overall decrease in the unpaired GC value. For example, at the transitions from RNA #1 to #2 and RNA #6 to #7, the unpaired GC value around the mutation sites decreased approximately 1.5 and 1, respectively (Fig. 6A), which contributed to 65% and 100% of the change of the unpaired GC value of the entire structure (Fig. 3). These results indicate that most of the mutations introduced in the course of evolution tend to alter the local structures. This tendency is reasonable because, during evolution, selected mutations are expected to improve defective structures without altering other nondefective structures. Second, the structural changes caused by mutations differed depending on the types of mutations: point mutation, deletion, and insertion. At the transitions where point mutations were introduced (i.e., RNA #1 to #2, #2 to #3, #5 to #6, and #8 to #9), new base pairs were generated to elongate or form stem structures without destroying existing stem structures (Fig. 5). In contrast, at the transitions where three-base deletions or a six-base insertion was introduced (i.e., RNA #3 to #4, #4 to #5, and #6 to #7), base-pairing patterns of existing stem structures significantly changed. For example, at the transition from RNA #3 to #4, the stem structure at base numbers 1996–2030 of RNA #3 was destabilized. At the transition from RNA #4 to #5, the stem structures at base numbers 1967–2012 of RNA #4 were deformed. At the transition from RNA #6 to #7, two small stem structures at base numbers 130–153 and 2073–2075 were reshaped. These results indicate that deletion and insertion events may induce large reconstructions of RNA structures.

The pattern of mutational sites also provides a useful insight for designing replicable ssRNA more efficiently. The nine out of 11 mutations analyzed here were accumulated in the structural regions A, B, and C, consisting of only 228 bases in total (base numbers 130–215, 740–765, and 1965–2080, Supplemental Fig. S4), indicating that these regions were the primal target for improvement during the evolution. The accumulation of mutations in these regions is reasonable because this region is the intersection of two different RNAs. We constructed RNA #1 by inserting the Qβ replicase subunit gene into a small (222 base) replicable RNA, MDV-1 (Mills et al. 1973). Although the insertion of a gene into MDV-1 has been widely used to make replicable ssRNAs by Qβ replicase (Miele et al. 1983; Lizardi et al. 1988; Wu et al. 1992), the insertion could destruct the optimized structures in MDV-1 and the replicase subunit gene. The result, the accumulation of mutations in the intersection, suggests that the replicability of ssRNA could be improved more efficiently by focusing on the structures around intersections.

## MATERIALS AND METHODS

### RNA preparation

The nine RNAs were prepared by in vitro transcription using corresponding plasmids as previously described (Usui et al. 2013). The plasmid for RNA#1 is the original plasmid, pUCmdv-WHL+, used in a previous study (Ichihashi et al. 2013). The other plasmids were constructed by introducing each mutation shown in Figure 1B by PCR with primers that each have a mutation, followed by self-ligation with the In-Fusion Cloning kit (Takara) and transformation into an *E. coli* strain (DH5α).

### RNA replication

RNA replication was performed with a template RNA (100 nM) and purified Qβ replicase (20 nM) at 37°C for 20 min in the presence of [^32^P]-UTP in a solution as previously described (Usui et al. 2013). The reaction mixtures were quenched in the sampling buffer (50 mM Tris-HCl [pH 7.4], 840 mM 2-mercaptoethanol, 2% sodium dodecyl sulfate [SDS], and 10% glycerol), and immediately subjected to 1% agarose gel electrophoresis in 1× TBE buffer at 4°C. After agarose-gel electrophoresis and autoradiography, the bands corresponding to ssRNAs and dsRNAs were measured. The template activity was calculated as the ssRNA concentrations, whereas the dsRNA ratio was calculated as the dsRNA concentrations divided by the sum of ssRNA and dsRNA concentrations.

### SHAPE analysis

SHAPE data was obtained as previously described (Mizuuchi et al. 2015) with the following modifications. We additionally used FAM-labeled primers for the sequencing reaction in the presence of ddATP to make the alignment more precise. The alignment was adjusted manually throughout the sequence after automatic alignment with QuShape software (Karabiber et al. 2013), and the regions where the alignment was uncertain due to unclear signal (∼10%) were removed from the data used for the following analysis. The average SHAPE values were normalized for each primer among the nine RNAs. SHAPE values less than zero and more than one were adjusted to zero or one, respectively. We performed SHAPE analysis three times throughout the sequence of the nine RNAs and used the average values for structure prediction. The average values and standard deviations are shown in the Supplemental Information file.

### Structure prediction

The partition function of each RNA was calculated using RNAstructure software (Mathews 2014) with the SHAPE values as a constraint. The parameters (slope = 2.6 and intercept = −0.8) were used for calculating the partition function (Deigan et al. 2009). The partition functions were used to (i) obtain dot plots, which display probabilities of any pairs in a sequence, and (ii) predict MEA structures using the Max Expect algorithm of the software (Mathews 2014; Mathews et al. 2009). The probabilities in the dot plots were used to calculate the unpaired GC value, the sum of the probability that each G or C nucleotide is not base-paired throughout the entire sequence of each RNA.

## SUPPLEMENTAL MATERIAL

Supplemental material is available for this article.

## Supplementary Material

Supplemental Material

## References

[RNA073106MIZC1] Attwater J, Raguram A, Morgunov AS, Gianni E, Holliger P. 2018 Ribozyme-catalysed RNA synthesis using triplet building blocks. Elife 7: e35355 10.7554/eLife.35255 PMC6003772 29759114

[RNA073106MIZC2] Axelrod VD, Brown E, Priano C, Mills DR. 1991 Coliphage Qβ RNA replication: RNA catalytic for single-strand release. Virology 184: 595–608. 10.1016/0042-6822(91)90430-J 1887587

[RNA073106MIZC3] Bansho Y, Furubayashi T, Ichihashi N, Yomo T. 2016 Host-parasite oscillation dynamics and evolution in a compartmentalized RNA replication system. Proc Natl Acad Sci 113: 4045–4050. 10.1073/pnas.1524404113 27035976 PMC4839452

[RNA073106MIZC4] Biebricher CK, Luce R. 1993 Sequence analysis of RNA species synthesized by Qβ replicase without template. Biochemistry 32: 4848–4854. 10.1021/bi00069a021 7683911

[RNA073106MIZC5] Brown D, Gold L. 1995 Selection and characterization of RNAs replicated by Q.beta. replicase. Biochemistry 34: 14775–14782. 10.1021/bi00045a019 7578086

[RNA073106MIZC6] Chetverin AB. 2018 Thirty years of studies of Qβ replicase: what have we learned and what is yet to be learned? Biochemistry 83: S19–S32. 29544428 10.1134/S0006297918140031

[RNA073106MIZC7] Chetverin AB, Chetverina HV, Demidenko AA, Ugarov VI. 1997 Nonhomologous RNA recombination in a cell-free system: evidence for a transesterification mechanism guided by secondary structure. Cell 88: 503–513. 10.1016/S0092-8674(00)81890-5 9038341 PMC7173214

[RNA073106MIZC8] Deigan KE, Li TW, Mathews DH, Weeks KM. 2009 Accurate SHAPE-directed RNA structure determination. Proc Natl Acad Sci 106: 97–102. 10.1073/pnas.0806929106 19109441 PMC2629221

[RNA073106MIZC9] Gytz H, Mohr D, Seweryn P, Yoshimura Y, Kutlubaeva Z, Dolman F, Chelchessa B, Chetverin AB, Mulder FAA, Brodersen DE, 2015 Structural basis for RNA-genome recognition during bacteriophage Qβ replication. Nucleic Acids Res 43: 10893–10906. 10.1093/nar/gkv1212 26578560 PMC4678825

[RNA073106MIZC10] Higgs PG, Lehman N. 2015 The RNA World: molecular cooperation at the origins of life. Nat Rev Genet 16: 7–17. 10.1038/nrg3841 25385129

[RNA073106MIZC11] Ichihashi N. 2019 What can we learn from the construction of in vitro replication systems? Ann N Y Acad Sci 1447: 144–156. 10.1111/nyas.14042 30957237

[RNA073106MIZC12] Ichihashi N, Usui K, Kazuta Y, Sunami T, Matsuura T, Yomo T. 2013 Darwinian evolution in a translation-coupled RNA replication system within a cell-like compartment. Nat Commun 4: 2494 10.1038/ncomms3494 24088711

[RNA073106MIZC13] Ichihashi N, Aita T, Motooka D, Nakamura S, Yomo T. 2015 Periodic pattern of genetic and fitness diversity during evolution of an artificial cell-like system. Mol Biol Evol 32: 3205–3214. 10.1093/molbev/msv189 26342111

[RNA073106MIZC14] Joyce GF, Szostak JW. 2018 Protocells and RNA self-replication. Cold Spring Harb Perspect Biol 10: a034801 10.1101/cshperspect.a034801 30181195 PMC6120706

[RNA073106MIZC15] Karabiber F, McGinnis JL, Favorov OV, Weeks KM. 2013 QuShape: rapid, accurate, and best-practices quantification of nucleic acid probing information, resolved by capillary electrophoresis. RNA 19: 63–73. 10.1261/rna.036327.112 23188808 PMC3527727

[RNA073106MIZC16] Kuppers B, Sumper M. 1975 Minimal requirements for template recognition by bacteriophage Q replicase: approach to general RNA-dependent RNA synthesis. Proc Natl Acad Sci 72: 2640–2643. 10.1073/pnas.72.7.2640 1058479 PMC432825

[RNA073106MIZC17] Lincoln TA, Joyce GF. 2009 Self-sustained replication of an RNA enzyme. Science 323: 1229–1232. 10.1126/science.1167856 19131595 PMC2652413

[RNA073106MIZC18] Lizardi P, Guerra C, Lomeli H, Tussie-Luna I, Kramer F. 1988 Exponential amplification of recombinant-RNA hybridization probes. Bio/Technology 6: 1197–1202. 10.1038/nbt1088-1197

[RNA073106MIZC19] Mathews DH. 2014 RNA secondary structure analysis using RNAstructure. Curr Protoc Bioinformatics 46: 12.6.1–12.6.25. 10.1002/0471250953.bi1206s13 24939127

[RNA073106MIZC20] Mathews DH, Lu ZJ, Gloor JW. 2009 Improved RNA secondary structure prediction by maximizing expected pair accuracy. RNA 15: 1805–1813. 10.1261/rna.1643609 19703939 PMC2743040

[RNA073106MIZC21] Merino EJ, Wilkinson KA, Coughlan JL, Weeks KM. 2005 RNA structure analysis at single nucleotide resolution by selective 2′-hydroxyl acylation and primer extension (SHAPE). J Am Chem Soc 127: 4223–4231. 10.1021/ja043822v 15783204

[RNA073106MIZC22] Miele EA, Mills DR, Kramer FR. 1983 Autocatalytic replication of a recombinant RNA. J Mol Biol 171: 281–295. 10.1016/0022-2836(83)90094-3 6655695

[RNA073106MIZC23] Mills DR, Kramer FR, Spiegelman S. 1973 Complete nucleotide sequence of a replicating RNA molecule. Science 180: 916–927. 10.1126/science.180.4089.916 4706684

[RNA073106MIZC24] Mills DR, Dobkin C, Kramer FR. 1978 Template-determined, variable rate of RNA chain elongation. Cell 15: 541–550. 10.1016/0092-8674(78)90022-3 719752

[RNA073106MIZC25] Mizuuchi R, Ichihashi N. 2018 Sustainable replication and coevolution of cooperative RNAs in an artificial cell-like system. Nat Ecol Evol 2: 1654–1660. 10.1038/s41559-018-0650-z 30150742

[RNA073106MIZC26] Mizuuchi R, Ichihashi N, Usui K, Kazuta Y, Yomo T. 2015 Adaptive evolution of an artificial RNA genome to a reduced ribosome environment. ACS Synth Biol 4: 292–298. 10.1021/sb5000884 24933578

[RNA073106MIZC27] Shimizu Y, Inoue A, Tomari Y, Suzuki T, Yokogawa T, Nishikawa K, Ueda T. 2001 Cell-free translation reconstituted with purified components. Nat Biotechnol 19: 751–755. 10.1038/90802 11479568

[RNA073106MIZC28] Szostak JW, Bartel DP, Luisi PL. 2001 Synthesizing life. Nature 409: 387–390. 10.1038/35053176 11201752

[RNA073106MIZC29] Takeshita D, Yamashita S, Tomita K. 2014 Molecular insights into replication initiation by Qβ replicase using ribosomal protein S1. Nucleic Acids Res 42: 10809–10822. 10.1093/nar/gku745 25122749 PMC4176380

[RNA073106MIZC30] Ueda K, Mizuuchi R, Matsuda F, Ichihashi N. 2019 A fusion method to develop an expanded artificial genomic RNA replicable by Qβ replicase. Chembiochem 20: 2331–2335. 10.1002/cbic.201900120 31037814

[RNA073106MIZC31] Ugarov VI, Chetverin AB. 2008 Functional circularity of legitimate Qβ replicase templates. J Mol Biol 379: 414–427. 10.1016/j.jmb.2008.03.074 18466922 PMC7173182

[RNA073106MIZC32] Ugarov VI, Demidenko AA, Chetverin AB. 2003 Qβ replicase discriminates between legitimate and illegitimate templates by having different mechanisms of initiation. J Biol Chem 278: 44139–44146. 10.1074/jbc.M305992200 12947121

[RNA073106MIZC33] Usui K, Ichihashi N, Kazuta Y, Matsuura T, Yomo T. 2013 Kinetic model of double-stranded RNA formation during long RNA replication by Qβ replicase. FEBS Lett 587: 2565–2571. 10.1016/j.febslet.2013.06.033 23831021

[RNA073106MIZC34] Usui K, Ichihashi N, Yomo T. 2015 A design principle for a single-stranded RNA genome that replicates with less double-strand formation. Nucleic Acids Res 43: 8033–8043. 10.1093/nar/gkv742 26202975 PMC4652763

[RNA073106MIZC35] Vaidya N, Manapat ML, Chen IA, Xulvi-Brunet R, Hayden EJ, Lehman N. 2012 Spontaneous network formation among cooperative RNA replicators. Nature 491: 72–77. 10.1038/nature11549 23075853

[RNA073106MIZC36] Vasilyev NN, Kutlubaeva ZS, Ugarov VI, Chetverina HV, Chetverin AB. 2013 Ribosomal protein S1 functions as a termination factor in RNA synthesis by Qβ phage replicase. Nat Commun 4: 1781 10.1038/ncomms2807 23653193

[RNA073106MIZC37] Wu Y, Zhang DY, Kramer FR. 1992 Amplifiable messenger RNA. Proc Natl Acad Sci 89: 11769–11773. 10.1073/pnas.89.24.11769 1465396 PMC50638

