# Supplementary material for: Structural transition of replicable RNAs during in vitro evolution with Qβ replicase
Source: RNA. 2020 Jan;26(1):83–90. doi: 10.1261/rna.073106.119 (PMC6913131; doi:10.1261/rna.073106.119)
Supplement: Supplemental Material [file supp_26_1_83__index.html]

Structural transition of replicable RNAs during in vitro evolution with Qβ replicase — Structural transition of replicable RNAs during in vitro evolution with Qβ replicase — Supplemental Material 

# Structural transition of replicable RNAs during in vitro evolution with Qβ replicase

## Supplemental Material

- Supplemental\_Figures\_and\_Text.docx
- Supplemental\_Information.xlsx
