# Supplementary material for: Structural transition of replicable RNAs during in vitro evolution with Qβ replicase
Source: RNA. 2020 Jan;26(1):83–90. doi: 10.1261/rna.073106.119 (PMC6913131; doi:10.1261/rna.073106.119)
Supplement: Supplemental Material [file supp_073106.119_Supplemental_Figures_and_Text.docx]

Supplemental Figures and Text

Structural transition of replicable RNAs during in vitro evolution with Qβ replicase

Ryo Mizuuchi, Kimihito Usui, Norikazu Ichihashi


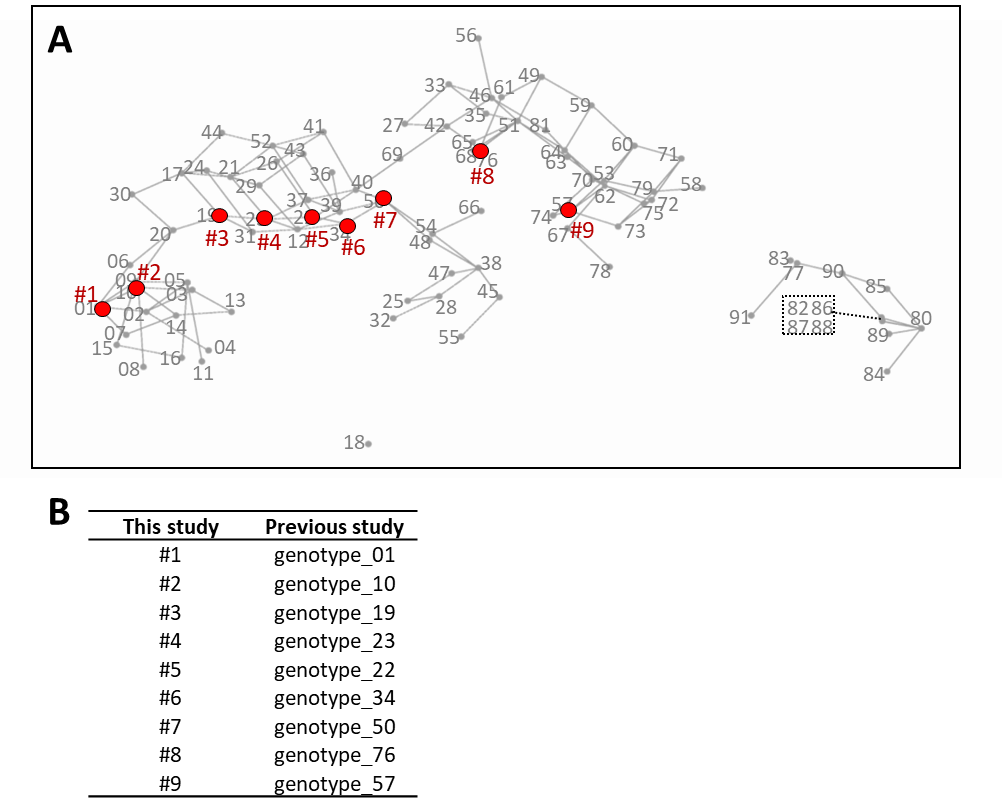


**Figure S1. Nine RNAs used in this study.**

(A) The locations of the nine RNAs in the genetic map presented in our previous study (Ichihashi et al. 2015). (B) The names of the nine RNAs in the previous study.


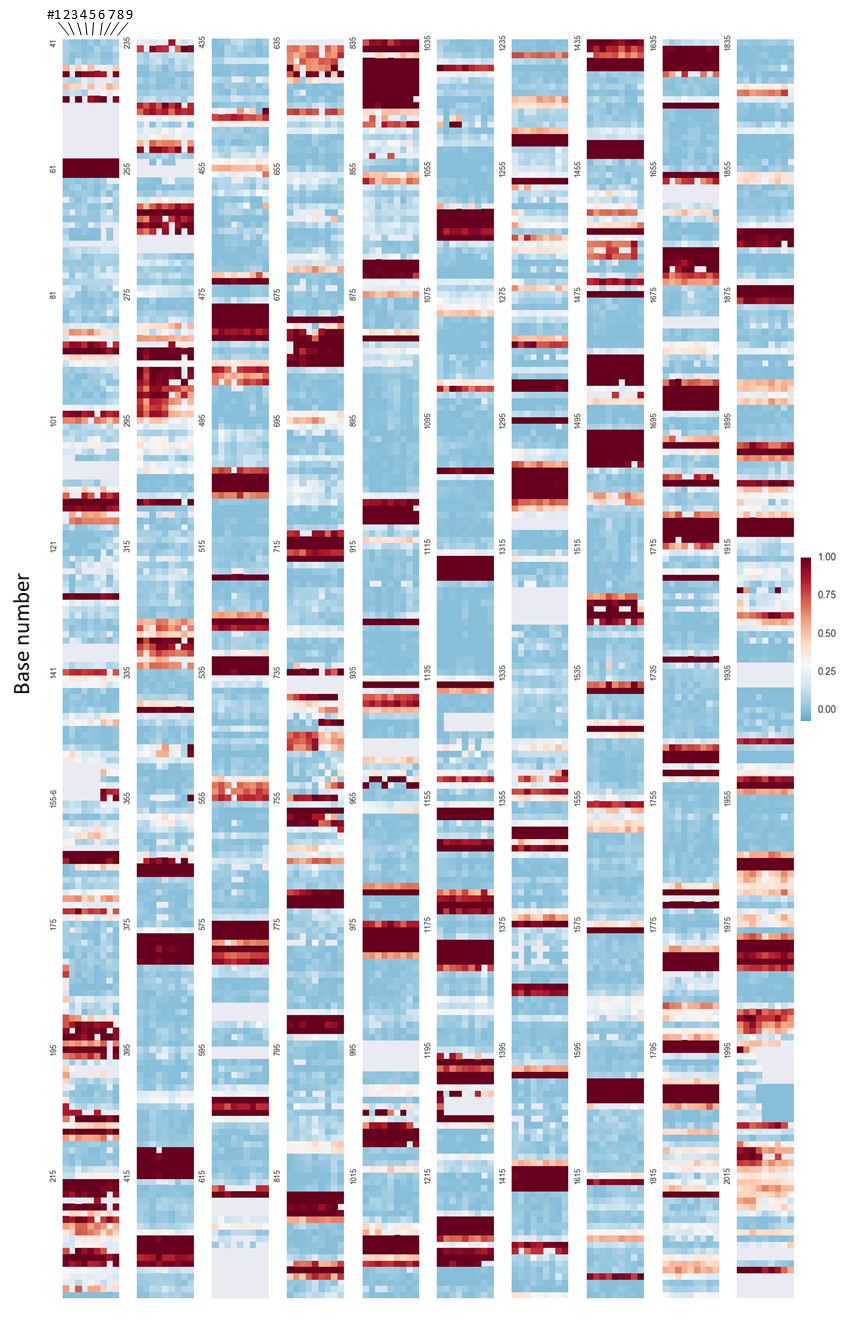


**Figure S2. SHAPE values of the nine RNAs.**

SHAPE data of approximately 90% of the entire sequences were obtained for all RNAs. The data in gray regions were not obtained due to unclear signal. Raw data is shown in the Supplemental file.


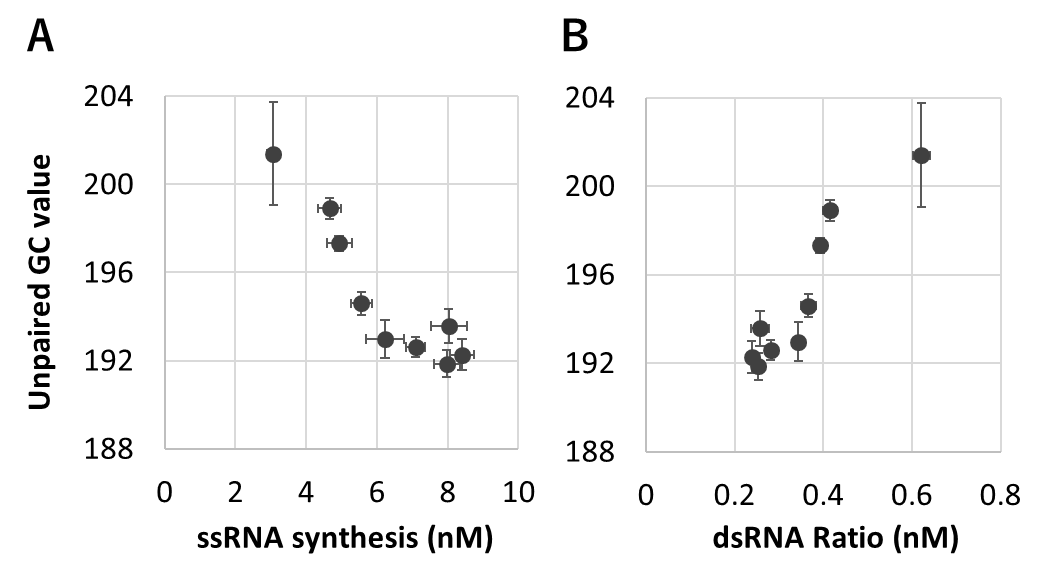


**Figure S3. Comparison of unpaired GC values with ssRNA synthesis and dsRNA ratio.**

The unpaired GC values (Fig. 3) were plotted against (A) the synthesized ssRNA concentration and (B) the dsRNA ratio in the replication experiment (Fig. 2A, B). The correlation coefficients are $-$0.92 and 0.93, respectively. The ssRNA synthesis reflects the template ability of each clone RNA.


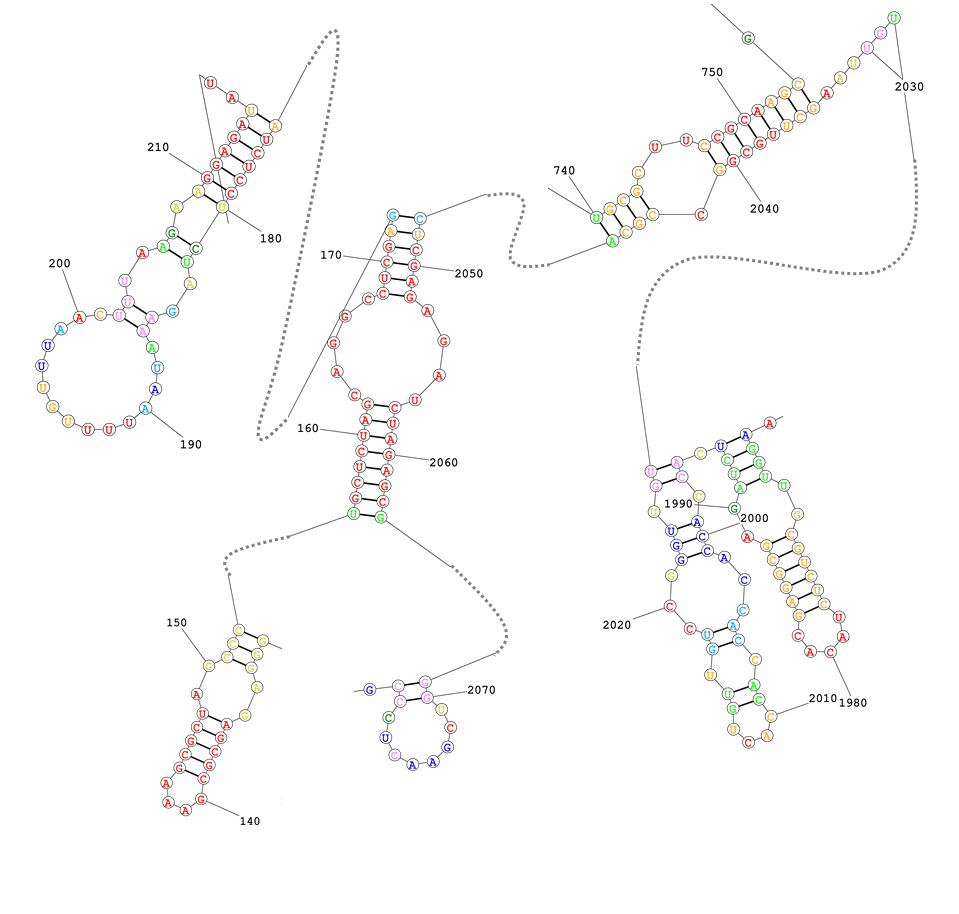


**Figure S4. The structural region comprising the regions A, B, and C.**

The MEA structure of RNA#1 is shown. Dotted lines represent 0–4 consecutive unpaired nucleotides.


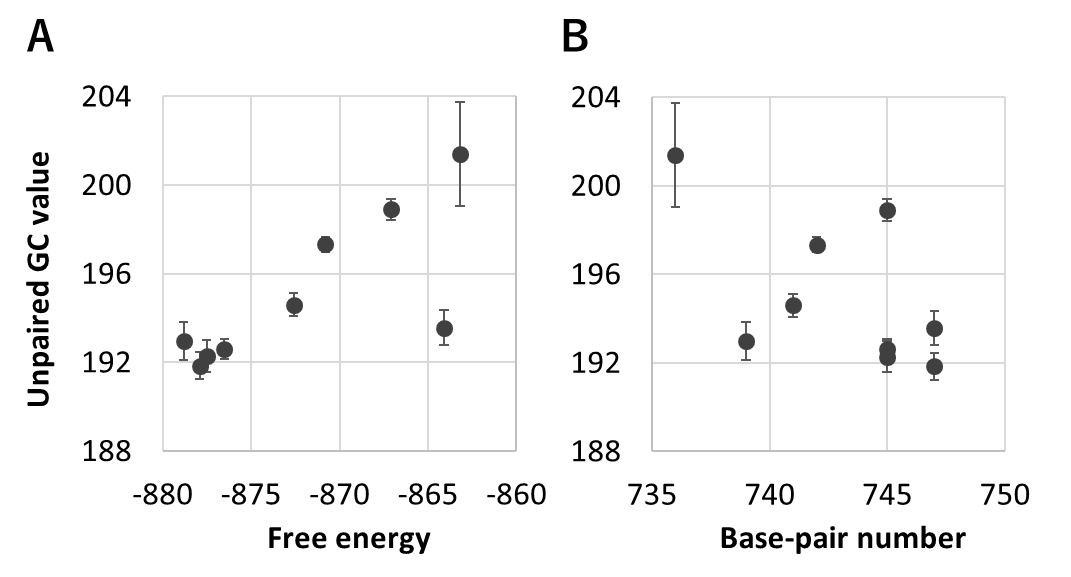


**Figure S5. Biophysical parameters.**

(A) The free energy of the overall structure for each MEA structure was calculated by the Efn2 algorithm of the same RNAstructure software and plotted against the unpaired GC values (Fig. 3). The correlation coefficient is $-$0.76. (B) The number of base pairs in each MEA structure, plotted against the unpaired GC values. The correlation coefficient is 0.58

**Supplemental text: MEA structures in bracket format.**

RNA#1

(((.(((((((...)))))))..((((((((((....)))))))).)).((((((((((...)))))))))).....((((((((.......)))))))).(((.(((((((.....))))))).))).(((..(((((....)))))..))).((((((((.....(((((((((((((((..((...............))..)).)))))))........(((...)))......((((((...(((.....(((((....))))).))).))))))............(.(((((((..((((.((((...((((.(((((((......)))))))..))))..(((((.(((....(((.(.(((((((((.....))))))))))))).(((((((.((...(((....(((((((((.....))))))))))))...)).)))))))..))).)))))(((((((.(((......)))....((((((((.((((.....)))))))))))))))))))..))))..(((((.(((..(((((((...(((.((.((((((((((((.......))))))))).(((((((((((....))))))))))).......))).)).)))....)))))))...))).))))).)))).(((((...(((((((........)))))))...)))))(((.((.(((((....))))).)).)))))))))).).((((...((((((((...........(((((..(((((.(((...(((((...)))))...))).))))).((((((((...)))))))).))))).............(((((((((((((((..(((((..((.((((((.(((.(((((((((((((((((((((.......)))))))))))).)))))))))...))))))))).)).(((.((((((..(((((.(((((....))))))))))..)))))).))).(((((..((((.(((((.((((....)))).)))))..((.(((.(((.((((((.(((.....))).))))))..))).))).))..((((((((.(((.(((((((((((((....))))))))))))))))..)))))))).)))).((((..(((..((((((((..(((((....))))).))))))))...))).....(((((..(((..((........))..)))..)))))..))))..))))).)))))..)))))(((((....(((..(((((..((((.((((.((......)).))))))))))))).)))(((((.((((((((((((.((((..((((((....))))))))))..)))))))))..))).)))))(((.((((((((.((((.....)))).))).)))))))).....(((((((((((...((((((((........(((.(.(((((((((........((((......))))...))))))))))))).......)))))))).(((((.(((((((((((((((.((((((.((((.(((.(((((.((.......)).)))))....))).)))).))).)))))))).))))).)))))....)))))((((((((((..(((.((((((......)))))).))).(((((....))))).))))).)))))....))))).((((((((((((.(((..(((((((((....)))))))))..))))))).).)))))))..)))))).((((...(((((...)))))))))))))).(((((.(((...(((.(((((((...((((.((((((((((((((....))))))..)))))))).))))....)))))))..)))...)))...)))))))))))))))((((((((((.((((....))))...)))))))))).((((..((((((.....))))))..)))).((.(((...((.((....)).))...))).))......)))))))).))))))))))....))))))))....((........))..((((((((....))))))))(((((((....))))))).))).

RNA#2

(((.(((((((...)))))))..((((((((((....)))))))).)).((((((((((...)))))))))).....((((((((.......)))))))).(((.(((((((.....))))))).))).(((..(((((....)))))..))).((((((((.....(((((((((((((((((((.(((......))).))))).).))))))).......((((...)))).....((((((.......(((.(((((....))))).))).))))))............(.(((((((..((((.((((...((((.(((((((......)))))))..))))..(((((.(((....(((.(.(((((((((.....))))))))))))).(((((((.((...(((....(((((((((.....))))))))))))...)).)))))))..))).)))))(((((((.((((....))))....((((((((.((((.....)))))))))))))))))))..))))..(((((.(((..(((((((...(((.((.((((((((((((.......))))))))).(((((((((((....))))))))))).......))).)).)))....)))))))...))).))))).)))).(((((...(((((((........)))))))...)))))(((.((.(((((....))))).)).)))))))))).).((((...((((((((.......((..(((((..(((((.(((...(((((...)))))...))).))))).((((((((...)))))))).))))).............(((((((((((((((..(((((..((.((((((.(((.(((((((((((((((((((((.......)))))))))))).)))))))))...))))))))).)).(((.((((((..(((((.(((((....))))))))))..)))))).))).(((((..((((.(((((.((((....)))).)))))..((.(((.(((.((((((.(((.....))).))))))..))).))).))..((((((((.(((.(((((((((((((....))))))))))))))))..)))))))).)))).((((..((((.((((((((..(((((....))))).))))))))....))))...(((((..(((..((........))..)))..)))))..))))..))))).)))))..)))))(((((....(((..(((((..((((.((((.((......)).))))))))))))).)))(((((.((((((((((((.((((..((((((....))))))))))..)))))))))..))).)))))(((.((((((((.((((.....)))).))).)))))))).....(((((((((((...((((((((........(((.(.(((((((((........((((......))))...))))))))))))).......)))))))).(((((.(((((((((((((((.((((((.((((.(((.(((((.((.......)).)))))....))).)))).))).)))))))).))))).)))))....)))))((((((((((..(((.((((((......)))))).))).(((((....))))).))))).)))))....))))).((((((((((((.(((..(((((((((....)))))))))..))))))).).)))))))..)))))).((((...(((((...)))))))))))))).(((((.(((...(((.(((((((...((((.((((((((((((((....))))))..)))))))).))))....)))))))..)))...)))...)))))))))))))))((((((((((.((((....))))...))))))))))..))...((((((.....)))))).......((.((.(((((.((....)).))...))).)).))...)))))))).))))))))))....))))))))...(((........))).((((((((....))))))))(((((((....))))))).))).

RNA#3

(((.(((((((...)))))))..((((((((((....)))))))).)).((((((((((...)))))))))).....((((((((.......)))))))).(((.(((((((.....))))))).))).(((..(((((....)))))..))).((((((((....((((((((((((((((((((.(((......))).))))).).)))))))........(((...)))......((((((.......(((.(((((....))))).))).))))))............(.(((((((..((((.((((...((((.(((((((......)))))))..))))..(((((.(((....(((.(.(((((((((.....))))))))))))).(((((((.((...(((....(((((((((.....))))))))))))...)).)))))))..))).)))))(((((((.((((....))))....((((((((.((((.....)))))))))))))))))))..))))..(((((.(((..(((((((...(((.((.((((((((((((.......))))))))).(((((((((((....))))))))))).......))).)).)))....)))))))...))).))))).)))).(((((...(((((((........)))))))...)))))(((.((.(((((....))))).)).)))))))))).).((((...((((((((.....(((((((((((..(((((.(((...(((((...)))))...))).))))).((((((((...)))))))).)))))..........((.(((((((((((((((..(((((..((.((((((.(((.(((((((((((((((((((((.......)))))))))))).)))))))))...))))))))).)).(((.((((((..(((((.(((((....))))))))))..)))))).))).(((((..((((.(((((.(((......))).)))))..((.(((.(((.((((((.(((.....))).))))))..))).))).))..((((((((.(((.(((((((((((((....))))))))))))))))..)))))))).)))).((((..((((.((((((((..(((((....))))).))))))))....))))...(((((..(((..((........))..)))..)))))..))))..))))).)))))..)))))(((((....(((..(((((..((((.((((.((......)).))))))))))))).)))(((((.((((((((((((.(((((..(((((....))))))))))..)))))))))..))).)))))(((.((((((((.((((.....)))).))).)))))))).....(((((((((((...((((((((........(((.(.(((((((((........((((......))))...))))))))))))).......)))))))).(((((.(((((((((((((((.((((((.((((.(((.(((((.((.......)).)))))....))).)))).))).)))))))).))))).)))))....)))))((((((((((..(((.((((((......)))))).))).(((((....))))).))))).)))))....))))).((((((((((((.(((..(((((((((....)))))))))..))))))).).)))))))..)))))).((((...(((((...)))))))))))))).(((((.(((...(((.(((((((...((((.((((((((((((((....))))))..)))))))).))))....)))))))..)))...)))...)))))))))))))))((((((((((.((((....))))...))))))))))......))..........)))))).......((.((.(((...((.......))...))).)).))...)))))))).)))))))))))...))))))))...(((........))).((((((((....))))))))(((((((....))))))).))).

RNA#4

(((.(((((((...)))))))..((((((((((....)))))))).)).((((((((((...)))))))))).....((((((((.......)))))))).(((.(((((((.....))))))).))).(((..(((((....)))))..))).((((((((....((((((((((((((((((((.(((......))).))))).).)))))))........(((...)))......((((((.......(((.(((((....))))).))).))))))............(.(((((((..((((.((((...((((.(((((((......)))))))..))))..(((((.(((....(((.(.(((((((((.....))))))))))))).(((((((.((...(((....(((((((((.....))))))))))))...)).)))))))..))).)))))(((((((.(((......)))....((((((((.(((((...))))))))))))))))))))..))))..(((((.(((..(((((((...(((.((.((((((((((((.......))))))))).(((((((((((....))))))))))).......))).)).)))....)))))))...))).))))).)))).(((((...(((((((........)))))))...)))))(((.((.(((((....))))).)).)))))))))).).((((...((((((((....((((((.(((((..(((((.(((...(((((...)))))...))).))))).((((((((...)))))))).))))).............(((((((((((((((..(((((..((.((((((.(((.(((((((((((((((((((((.......)))))))))))).)))))))))...))))))))).)).(((.((((((..(((((.(((((....))))))))))..)))))).))).(((((..((((.(((((.((((....)))).)))))..((.(((.(((.((((((.(((.....))).))))))..))).))).))..((((((((.(((.(((((((((((((....))))))))))))))))..)))))))).)))).((((..((((.((((((((..(((((....))))).))))))))....))))...(((((..(((..((........))..)))..)))))..))))..))))).)))))..)))))(((((....(((..(((((..((((.((((.((......)).))))))))))))).)))(((((.((((((((((((.((((..((((((....))))))))))..)))))))))..))).)))))(((.((((((((.((((.....)))).))).)))))))).....(((((((((((...((((((((........(((.(.(((((((((........((((......))))...))))))))))))).......)))))))).(((((.(((((((((((((((.((((((.((((.(((.(((((.((.......)).)))))....))).)))).))).)))))))).))))).)))))....)))))((((((((((..(((.((((((......)))))).))).(((((....))))).))))).)))))....))))).((((((((((((.(((..(((((((((....)))))))))..))))))).).)))))))..)))))).((((...(((((...)))))))))))))).(((((.(((...(((.(((((((...((((.((((((((((((((....))))))..)))))))).))))....)))))))..)))...)))...)))))))))))))))((((((((((.((((....))))...))))))))))..((...((((((.....))))))...........))...((....)).....))).)))......)))))))).)))))))))))...))))))))....((........))..((((((((....))))))))(((((((....))))))).))).

RNA#5

(((.(((((((...)))))))..((((((((((....)))))))).)).((((((((((...)))))))))).....((((((((.......)))))))).(((.(((((((.....))))))).))).(((..(((((....)))))..))).((((((((....((((((((((((((((((((.(((......))).))))).).)))))))........(((...)))......((((((.......(((.(((((....))))).))).))))))............(.(((((((..((((.((((...((((.(((((((......)))))))..))))..(((((.(((....(((.(.(((((((((.....))))))))))))).(((((((.((...(((....(((((((((.....))))))))))))...)).)))))))..))).)))))(((((((.(((......)))....((((((((.((((.....)))))))))))))))))))..))))..(((((.(((..(((((((...(((.((.((((((((((((.......))))))))).(((((((((((....))))))))))).......))).)).)))....)))))))...))).))))).)))).(((((...(((((((........)))))))...)))))(((.((.(((((....))))).)).)))))))))).).((((...((((((((....((((((.((((...(((((.(((...(((((...)))))...))).))))).((((((((...))))))))..)))).............(((((((((((((((..(((((..((.((((((.(((.(((((((((((((((((((((.......)))))))))))).)))))))))...))))))))).)).(((.((((((..(((((.(((((....))))))))))..)))))).))).(((((..((((.(((((.((((....)))).)))))..((.(((.(((.((((((.(((.....))).))))))..))).))).))..((((((((.(((.(((((((((((((....))))))))))))))))..)))))))).)))).((((..((((.((((((((..(((((....))))).))))))))....))))...(((((..(((..((........))..)))..)))))..))))..))))).)))))..)))))(((((....(((..(((((..((((.((((.((......)).))))))))))))).)))(((((.((((((((((((.((((..((((((....))))))))))..)))))))))..))).)))))(((.((((((((.((((.....)))).))).)))))))).....(((((((((((...((((((((........(((.(.(((((((((........((((......))))...))))))))))))).......)))))))).(((((.(((((((((((((((.((((((.((((.(((.(((((.((.......)).)))))....))).)))).))).)))))))).))))).)))))....)))))((((((((((..(((.((((((......)))))).))).(((((....))))).))))).)))))....))))).((((((((((((.(((..(((((((((....)))))))))..))))))).).)))))))..)))))).((((...(((((...)))))))))))))).(((((.(((...(((.(((((((...((((.((((((((((((((....))))))..)))))))).))))....)))))))..)))...)))...)))))))))))))))((((((((((.((((....))))...))))))))))..(((..((((((.....))))))..))).....................))).)))......)))))))).)))))))))))...))))))))...(((........))).((((((((....))))))))(((((((....))))))).))).

RNA#6

(((.(((((((...)))))))..((((((((((....)))))))).)).((((((((((...)))))))))).....((((((((.......)))))))).(((.(((((((.....))))))).))).(((..(((((....)))))..))).((((((((....((((((((((((((((((((.(((......))).))))).).)))))))........(((...)))......((((((.......(((.(((((....))))).))).))))))............(.(((((((..((((.((((...((((.(((((((......)))))))..))))..(((((.(((....(((.(.(((((((((.....))))))))))))).(((((((.((...(((....(((((((((.....))))))))))))...)).)))))))..))).)))))(((((((.(((......)))....((((((((.((((.....)))))))))))))))))))..))))..(((((.(((..(((((((...(((.((.((((((((((((.......))))))))).(((((((((((....))))))))))).......))).)).)))....)))))))...))).))))).)))).(((((...(((((((........)))))))...)))))(((.((.(((((....))))).)).)))))))))).)..((....((((((((....((((((.(((((..(((((.(((...(((((...)))))...))).))))).((((((((...)))))))).))))).............(((((((((((((((..(((((..((.((((((.(((.(((((((((((((((((((((.......)))))))))))).)))))))))...))))))))).)).(((.((((((..(((((.(((((....))))))))))..)))))).))).(((((..((((.(((((.((((....)))).)))))..((.(((.(((.((((((.(((.....))).))))))..))).))).))..((((((((.(((.(((((((((((((....))))))))))))))))..)))))))).)))).((((..(((((((((((((..(((((....))))).)))))))))...))))...(((((..(((..((........))..)))..)))))..))))..))))).)))))..)))))(((((.((.(((..(((((..((((.((((.((......)).))))))))))))).)))(((((.((((((((((((.((((..((((((....))))))))))..)))))))))..))).)))))(((.((((((((.((((.....)))).))).)))))))).))..(((((((((((...((((((((........(((.(.(((((((((........((((......))))...))))))))))))).......)))))))).(((((.(((((((((((((((.((((((.((((.(((.(((((.((.......)).)))))....))).)))).))).)))))))).))))).)))))....)))))((((((((((..(((.((((((......)))))).))).(((((....))))).))))).)))))....))))).((((((((((((.(((..(((((((((....)))))))))..))))))).).)))))))..)))))).((((...(((((...)))))))))))))).(((((.(((...(((.(((((((...((((.((((((((((((((....))))))..)))))))).))))....)))))))..)))...)))...)))))))))))))))((((((((((.((((....))))...))))))))))..(((..((((((.....))))))..)))..((.((.......)).))..))).)))......))))))))..)).)))))))...))))))))...(((........))).((((((((....))))))))(((((((....))))))).))).

RNA#7

(((.(((((((...)))))))..((((((((((....)))))))).)).((((((((((...)))))))))).....((((((((.......)))))))).(((.(((((((.....))))))).))).((((((((((....))))).(.(((((.....(((((((....((((((((((((((((((((.(((......))).))))).).))))))).......((((...)))).....((((((.......(((.(((((....))))).))).))))))............(.(((((((..((((.((((...((((.(((((((......)))))))..))))..(((((.(((....(((.(.(((((((((.....))))))))))))).(((((((.((...(((....(((((((((.....))))))))))))...)).)))))))..))).)))))(((((((.(((......)))....((((((((.((((.....)))))))))))))))))))..))))..(((((.(((..(((((((...(((.((.((((((((((((.......))))))))).(((((((((((....))))))))))).......))).)).)))....)))))))...))).))))).)))).(((((...(((((((........)))))))...)))))(((.((.(((((....))))).)).)))))))))).)..((....((((((((....(((.(((((((...(((((.(((...(((((...)))))...))).))))).((((((((...))))))))..((............)).(((((((((((((((..(((((..((.((((((.(((.(((((((((((((((((((((.......)))))))))))).)))))))))...))))))))).)).(((.((((((..(((((.(((((....))))))))))..)))))).))).(((((..((((.(((((.((((....)))).)))))..((.(((.(((.((((((.(((.....))).))))))..))).))).))..((((((((.(((.(((((((((((((....))))))))))))))))..)))))))).)))).((((..(((((((((((((..(((((....))))).)))))))))...))))...(((((..(((..((........))..)))..)))))..))))..))))).)))))..)))))(((((....(((..(((((..((((.((((.((......)).))))))))))))).)))(((((.((((((((((((.(((((..(((((....))))))))))..)))))))))..))).)))))(((.((((((((.((((.....)))).))).)))))))).....(((((((((((...((((((((........(((.(.(((((((((........((((......))))...))))))))))))).......)))))))).(((((.(((((((((((((((.((((((.((((.(((.(((((.((.......)).)))))....))).)))).))).)))))))).))))).)))))....)))))((((((((((..(((.((((((......)))))).))).(((((....))))).))))).)))))....))))).((((((((((((.(((..(((((((((....)))))))))..))))))).).)))))))..)))))).((((...(((((...)))))))))))))).(((((.(((...(((.(((((((...((((.((((((((((((((....))))))..)))))))).))))....)))))))..)))...)))...)))))))))))))))((((((((((.((((....))))...))))))))))..(((..((((((.....)))))).......)))...((....)).))))))).)))......))))))))..)).)))))))...)))))))..))))))....)))))..((((((((....))))))))(((((((....))))))).))).

RNA#8

(((.(((((((...)))))))..((((((((((....)))))))).)).((((((((((...)))))))))).....((((((((.......)))))))).(((.(((((((.....))))))).))).((((((((((....))))).(.(((((.....(((((((....((((((((((((((((((((.(((......))).))))).).)))))))..............((((((...((((((.......(((.(((((....))))).))).))))))............(((((((((..((((.((((...((((.(((((((......)))))))..))))..(((((.(((....(((.(.(((((((((.....))))))))))))).(((((((.((...(((....(((((((((.....))))))))))))...)).)))))))..))).)))))((((((..(((......)))....((((((((.((((.....)))))))))))).))))))..))))..(((((.(((..(((((((...(((.((.((((((((((((.......))))))))).(((((((((((....))))))))))).......))).)).)))....)))))))...))).))))).)))).(((((...(((((((........)))))))...)))))(((.((.(((((....))))).)).)))))))))).(((((......)))))(......)))..(((((..(((((.(((...(((((...)))))...))).))))).((((((((...)))))))).))))).............(((((((((((((((..(((((..((.((((((.(((.(((((((((((((((((((((.......)))))))))))).)))))))))...))))))))).)).(((.((((((..(((((.(((((....))))))))))..)))))).))).(((((..((((.(((((.((((....)))).)))))..((.(((.(((.((((((.(((.....))).))))))..))).))).))..((((((((.(((.(((((((((((((....))))))))))))))))..)))))))).)))).((((..((((.((((((((..(((((....))))).))))))))....))))...(((((..(((..((........))..)))..)))))..))))..))))).)))))..)))))(((((....(((..(((((..((((.((((.((......)).))))))))))))).)))(((((.((((((((((((.(((((..(((((....))))))))))..)))))))))..))).)))))(((.((((((((.((((.....)))).))).)))))))).....(((((((((((...((((((((........(((.(.(((((((((........((((......))))...))))))))))))).......)))))))).(((((.(((((((((((((((.((((((.((((.(((.(((((.((.......)).)))))....))).)))).))).)))))))).))))).)))))....)))))((((((((((..(((.((((((......)))))).))).(((((....))))).))))).)))))....))))).((((((((((((.(((..(((((((((....)))))))))..))))))).).)))))))..)))))).((((...(((((...)))))))))))))).(((((.(((...(((.(((((((...((((.((((((((((((((....))))))..)))))))).))))....)))))))..)))...)))...)))))))))))))))((((((((((.((((....))))...)))))))))).((((..((((((.....))))))..)))).(((((.((....)).))...))).)))))).((((...))))...)))))))...)))))))..))))))....)))))..((((((((....))))))))(((((((....))))))).))).

RNA#9

(((.(((((((...)))))))..((((((((((....)))))))).)).((((((((((...)))))))))).....((((((((.......)))))))).(((.(((((((.....))))))).))).((((((((((....)))))...(((((.....(((((((....((((((((((((((((((((.(((......))).))))).).))))))).......((((...)))).....((((((...(((.....(((((....))))).))).))))))..........(((((((....)))))))((((...((((.(((((((......)))))))..))))..(((((.(((....(((.(.(((((((((.....))))))))))))).(((((((.((...(((....(((((((((.....))))))))))))...)).)))))))..))).)))))(((((((.(((......)))....((((((((.((((.....)))))))))))))))))))..))))...((((.(((..(((((((...(((.((.((((((((((((.......))))))))).(((((((((((....))))))))))).......))).)).)))....)))))))...))).))))(((((..(((((...(((((((........)))))))...)))))(((.((.(((((....))))).)).))))))))............((((((((.(.........(((((..(((((.(((...(((((...)))))...))).))))).((((((((...)))))))).))))).............(((((((((((((((..(((((..((.((((((.(((.(((((((((((((((((((((.......)))))))))))).)))))))))...))))))))).)).(((.((((((..(((((.(((((....))))))))))..)))))).))).(((((..((((.(((((.((((....)))).)))))..((.(((.(((.((((((.(((.....))).))))))..))).))).))..((((((((.(((.(((((((((((((....))))))))))))))))..)))))))).)))).((((..(((((((((((((..(((((....))))).)))))))))...))))...(((((..(((..((........))..)))..)))))..))))..))))).)))))..)))))(((((....(((..(((((..((((.((((.((......)).))))))))))))).)))(((((.((((((((((((.((((..((((((....))))))))))..)))))))))..))).)))))(((.((((((((.((((.....)))).))).)))))))).....(((((.(((((...((((((((..(..((((((.(.(((((((((........((((......))))...)))))))))))))))).)..)))))))).(((((.(((((((((((((((.((((((.((((.(((.(((((.((.......)).)))))....))).)))).))).)))))))).))))).)))))....)))))((((((((((..(((.((((((......)))))).))).(((((....))))).))))).)))))....))))).((((((((((((.(((..(((((((((....)))))))))..))))))).).)))))))...))))).((((...(((((...)))))))))))))).(((((.(((...(((.(((((((...((((.(((((((.((((((....))))))...))))))).))))....)))))))..)))...)))...)))))))))))))))((((((((((.((((....))))...)))))))))).((((..((((((.....))))))..)))).((.((.(((...........))).)).)).).)))))))).....)))))))...)))))))..))))).....)))))..((((((((....))))))))(((((((....))))))).))).
